# Standardized Protocols for Soil Fauna Extraction and a Call for Cross‐Lab Implementation

**DOI:** 10.1002/ece3.73407

**Published:** 2026-04-21

**Authors:** Michala Tůmová, Jing‐Zhong Lu, Maria J. I. Briones, Simone Cesarz, Nico Eisenhauer, Saori Fujii, Konstantin B. Gongalsky, Jari Haimi, Karin Hohberg, Stephan Jänsch, Hannah Karuri, Yudai Kitagami, Daniil I. Korobushkin, Alfred Lochner, Jérome Mathieu, Oksana L. Rozanova, Jörg Römbke, Julia Seeber, Rüdiger Schmelz, Olaf Schmidt, Clement Schneider, Xin Sun, Jiri Tuma, Andrey S. Zaitsev, Andrey G. Zuev, Anton M. Potapov

**Affiliations:** ^1^ Institute of Soil Biology and Biogeochemistry Biology Centre of the Czech Academy of Sciences České Budějovice Czech Republic; ^2^ Department of Soil Zoology Senckenberg Museum of Natural History Görlitz Görlitz Germany; ^3^ Departamento de Ecologia y Biologia Animal Universidade de Vigo Vigo Spain; ^4^ German Center for Integrative Biodiversity Research (iDiv) Leipzig Germany; ^5^ Institute of Biology Leipzig University Leipzig Germany; ^6^ Department of Forest Entomology Forestry and Forest Products Research Institute Tsukuba Japan; ^7^ A.N. Severtsov Institute of Ecology and Evolution Russian Academy of Sciences Moscow Russia; ^8^ Department of Biological and Environmental Science University of Jyväskylä Jyväskylä Finland; ^9^ ECT Oekotoxikologie GmbH Flörsheim Germany; ^10^ Department of Biological Sciences University of Embu Embu Kenya; ^11^ Laboratory of Forest Mycology Graduate School of Bioresources, Mie University Tsu Mie Japan; ^12^ Sorbonne Université, CNRS, IRD, INRAE, Université Paris Est Créteil, Université de Paris Cité, Institute of Ecology and Environmental Sciences of Paris (iEES‐Paris) Paris France; ^13^ Institute for Alpine Environment, Eurac Research Bozen/Bolzano Italy; ^14^ Department of Ecology Universität Innsbruck Innsbruck Austria; ^15^ Enchylab Martorell Spain; ^16^ School of Agriculture and Food Science University College Dublin Dublin 4 Ireland; ^17^ Key Laboratory of Urban Environment and Health, Ningbo Observation and Research Station, Institute of Urban Environment, Chinese Academy of Sciences Xiamen China; ^18^ Zhejiang Key Laboratory of Urban Environmental Processes and Pollution Control CAS Haixi Industrial Technology Innovation Center in Beilun Ningbo China; ^19^ International Institute Zittau TUD Dresden University of Technology Zittau Germany; ^20^ Johann Friedrich Blumenbach Institute of Zoology and Anthropology University of Göttingen Göttingen Germany

**Keywords:** invertebrates, macrofauna, mesofauna, microfauna, soil biodiversity, soil BON Foodweb, standardized methods

## Abstract

Understanding the status and global trends of soil invertebrate diversity requires accurate and comparable data across geographical regions. However, soil animal extraction approaches still vary among laboratories, and no commonly accepted, openly available and well‐documented protocols exist across taxa. Here, we present harmonized methodologies, assembled by an international group of experts, for extracting soil‐ and litter‐inhabiting nematodes, enchytraeids, microarthropods, and larger invertebrates. Illustrated with images and videos, the protocols include advice for overcoming the most frequently encountered issues (’expert tips’) for maximizing extraction efficiency and reproducibility. In addition, we provide results from two pilot experiments that test certain steps in nematode and large invertebrate extractions. We show that using two layers of milk filters instead of one in wet extraction of nematodes yields very similar extraction efficiency and has little effect on the sample cleanliness. Further, we demonstrate that on average 31.3% of large soil animals (body length 3 mm and longer) are overlooked during hand sorting but were captured by heat extraction of the sorted samples across three geographically distinct laboratories. Observed differences among the laboratories call for standardized tests of extraction efficiency of large soil animals across regions and research groups. Overall, we provide openly available expert protocols for assessing soil animal taxa in ecological studies worldwide, ultimately facilitating comparisons for a better understanding of the distribution and dynamics of soil biodiversity.

## Graphical abstract

We present harmonized methodologies, assembled by an international group of experts, for extracting soil‐ and litter‐inhabiting nematodes, enchytraeids, microarthropods, and larger invertebrates. The protocols are illustrated with images and videos, and include advice for overcoming the most frequent issues (’expert tips’) for increasing extraction efficiency and reproducibility. Openly available, these protocols can be used in ecological studies worldwide, facilitating comparisons for a better understanding of the dynamics of global soil biodiversity.

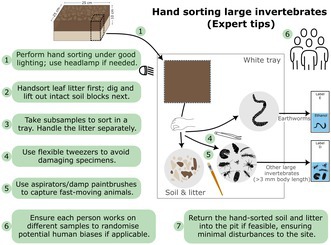

## 1 Introduction

Soil animals including micro‐, meso‐, and macrofauna play key roles in ecosystem functioning and services including food provision, water cleaning, and climate regulation (Lavelle and Spain 2002). Current changes in climate, land use, and pollution threaten soil animal diversity and related ecosystem functions (Geisen et al. 2019; FAO 2020). Therefore, monitoring soil animals is crucial to detecting potential changes. Recently, several initiatives to compile the distribution and abundance of soil animals at global scale have been accomplished (Fierer et al. 2009; Phillips et al. 2019; van den Hoogen et al. 2019; Lavelle et al. 2022; Potapov et al. 2023). Despite important insights, these studies have limitations, namely: (1) they consider different groups of organisms separately, (2) they provide snapshot data with very few temporal series available, and (3) almost all of them are based on a variety of sampling and extraction methods (Eisenhauer et al. 2020). The lack of unified monitoring and knowledge on the dynamics of soil animal diversity and abundance restrains the drafting of legislation on their protection (Guerra, Bardgett, et al. 2021). As a result, the main challenge remains to include multiple soil animal groups in a regular global monitoring and to use one harmonized methodology covering different types of habitats, including less studied areas of the world such as tropical regions (Cameron et al. 2019; Guerra et al. 2020).

Recently established, Soil BON Foodweb (SBF) is a global monitoring initiative collecting data on soil micro‐, meso‐, and macrofauna communities at a global scale using a unified methodology (Potapov et al. 2022, www.soilbonfoodweb.org). The main goals of the initiative are to explore drivers and functions of soil animal diversity, including interactions in soil food webs, and to assess the efficiency of current conservation measures for protection of soil animals at a global scale by following the Soil BON sampling design (Guerra, Wall, and Eisenhauer 2021). To ensure comparability of data coming from different laboratories, an expert team was established to summarize existing methodologies that can be applied across different countries, from polar regions to the tropics. The following requirements were pivotal for the selection of extraction methods: (1) low cost or DIY‐friendly, and easy‐to‐use equipment; (2) technical feasibility of deployment across main soil types and climatic zones, including remote sites; (3) possibility of biomass‐per‐area assessment (quantitative) and (4) possibility for subsequent diversity analysis of collected animals with molecular methods. The expert team deliberately focused on traditional animal extraction methods instead of environmental DNA (eDNA) sampling, since the former permits biomass quantification and avoids cross‐border transfer of soils for centralized analyses (therefore relying on local expertise and developing local capacity). Furthermore, recent EU‐scale assessments highlighted potentially strong methodological biases of eDNA methods, which apparently capture very different information on biodiversity than traditional ones (Köninger et al. 2025). While this supports the use of traditional methods, it is well documented that these vary in their efficiency among taxa (McSorley and Walter 1991). Therefore, four complementary extraction approaches—wet and dry funnel extractions and hand sorting—were adopted according to the target animal groups, as a basis for standardized monitoring of soil biodiversity by a global community of scientists interested in soil fauna and their cross‐taxa interactions.

**FIGURE 1:**
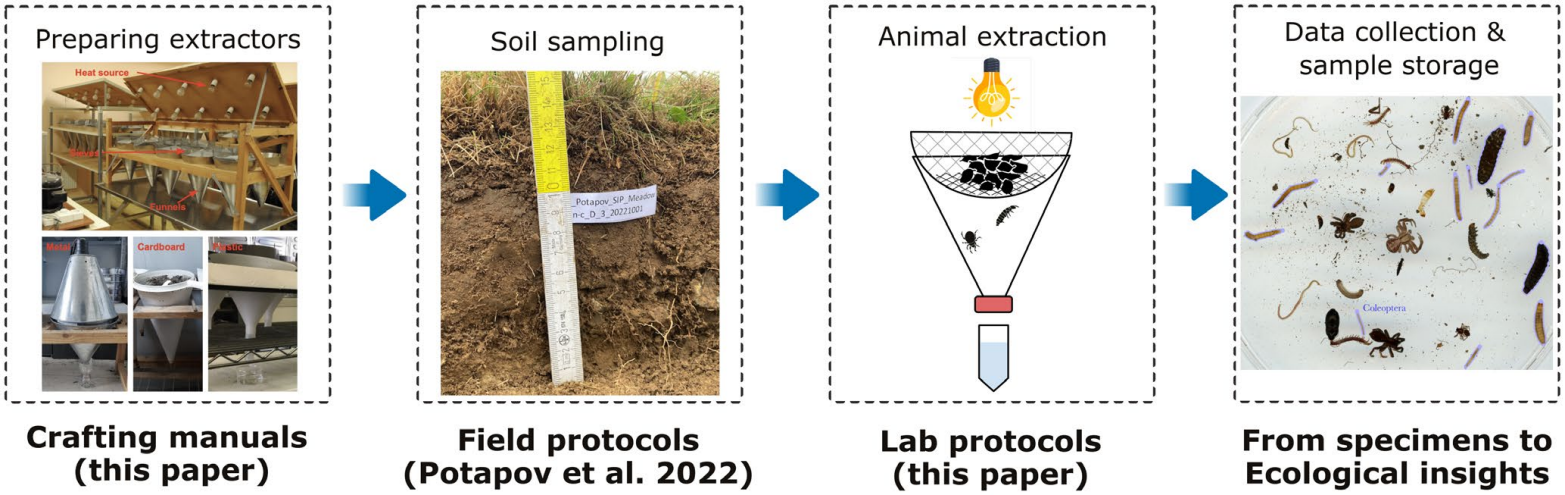
Flowchart showing the implementation for crafting manuals, field sampling, laboratory protocols for extracting soil animals, and downstream data collection.

Here, we briefly review existing extraction approaches and then detail the protocols, including video descriptions, of selected extraction methods for nematodes, enchytraeids, microarthropods, and larger invertebrates (see Zenodo repository). We explain the rationale for selecting a specific method, indicate its pros and cons, and describe steps critical to maximizing extraction efficiency, based on expert knowledge and consensus. Finally, we provide results from two experiments testing variations of extraction methods or evaluating extraction efficiency to refine some of our points. We finish with perspectives for further testing and development using the methods described here. The field protocols including common sampling protocols have been published earlier (Potapov et al. 2022). Our primary goal in this paper is to deliver openly available laboratory protocols (Figure 1), with detailed descriptions, maximizing reproducibility of soil animal extractions across the global monitoring network and other soil fauna assessments. With this, we have two major aims: (1) to increase awareness about poor standardization of laboratory extraction protocols among soil ecologists and provide practical solutions for this issue, and (2) to provide a single resource for methodologies of soil fauna assessment across size classes for ecological projects. The crafting manuals are provided for both wet and dry extractors (Zenodo repository: Files 01 and 02).

## 2 Nematode Extraction

Nematodes (phylum Nematoda) are worm‐like metazoans with body lengths of soil‐dwelling species ranging from 0.15 to 5 mm and body widths from 2 to 100 μm, which places them into the category of microfauna (Swift et al. 1979; Orgiazzi et al. 2015; Orgiazzi 2016). Many different methods are available to extract free‐living nematodes from soils and have been reviewed comprehensively (Nijs 2013). The main differences are in the extraction principle, such as live extraction based on their active movement (e.g., Baermann funnel), different sedimentation rate due to differences in density of soil particles versus nematodes (e.g., Oostenbrink elutriator), or separation based on the size of the nematode (e.g., Cobb sieves). None of these methods is perfect, and there are trade‐offs between efficiency, costs, sample size, duration, and cleanliness of the extracted sample (Nijs 2013). A globally useful method for nematode diversity requires avoiding complex devices, e.g., the Oostenbrink elutriator, and instead prioritizing simple methods that use inexpensive and widely available materials.

The Baermann Funnel method (Baermann 1917) with several adaptations (Verschoor and de Goede 2000; Cesarz et al. 2019; Ghaderi et al. 2025) requires only simple equipment and is the method of choice of the expert team. Soil with nematodes is spread on a milk filter (e.g., diameter 16–25 cm; Sana, Buchbach, Germany) on a coarse sieve, and is then placed in an empty funnel, which is then filled carefully with water. Within the next 72 h, the nematodes move actively downward through the soil, and then sink down passively in the free water body in the funnel. The nematodes are collected in a tube attached to the bottom of the funnel stem. Our recommended extraction time is three consecutive days (72 h) to include slow‐moving nematodes (Ghaderi et al. 2025). To counteract negative effects of possible losses due to predation, nematodes are collected and fixed in 24 h intervals by exchanging the vial with a fresh one and carefully adding some water from above (Data S1: Wet cold extractors). Since the extraction apparatus is filled with water and comes into contact with living organisms, we recommend using only non‐demineralized water of drinking quality.

The method is biased against certain nematode species that migrate upward rather than downward, e.g., *Bursaphelenchus cocophilus* and some insect parasites (Van Bezooijen 2006). Furthermore, this method selects against very slow or inactive nematodes (dauerlarvae and cryptobiotic state) and nematode eggs. Compared to other methods, the Baermann funnel technique is efficient in isolating nematodes from soils with high clay and organic matter content, giving a relatively clean sample. Increasing funnel size or soil layer thickness to accommodate larger samples increases the extraction duration and often compromises sample cleanliness and extraction efficiency.

The cleanliness of the sample, i.e., minimal amount of soil particles, is an important criterion in the Soil BON Foodweb, since it is essential for the subsequent use of image analysis techniques (Potapov et al. 2022). Large amounts of soil particles interfere with morphological identification, counting, and DNA extraction. Therefore, the use of a milk filter or a cheese cloth placed between soil sample and sieve is mandatory (Cesarz et al. 2019). However, these materials are not always easy to obtain and differ in their characteristics depending on the supplier. Based on cost considerations, milk filters were selected by the Soil BON Foodweb Team. The only disadvantage is that species with a highly excrescent cuticle (e.g., Criconematidae) are more likely to get stuck on the filter materials, leading to taxon‐specific bias (Ferris et al. 2004; Olson et al. 2017). The use of commercial paper towels is not recommended as it may reduce the extraction efficiency by more than 50% (Cesarz et al. 2019). A prerequisite for all sorts of filters is, however, the careful handling of the soil sample so that particles do not fall through during sample placement or submersion. To further improve cleanliness of the extraction and decrease the amount of soil particles in the collection vial, we tested the extraction efficiency using two layers of milk filters (see section *Pilot Experiment 1*).

Another aspect to consider is that the thickness of the soil layer (i.e., the height of soil in the funnel) and its aggregation significantly influence nematode extraction efficiency (Cesarz et al. 2019). Due to their small size, nematodes can only move small distances, so large amounts of soil, and more specifically, the soil height on the sieve, has a negative effect on the extraction efficiency (Van Bezooijen 2006; Nijs 2013; Cesarz et al. 2019). Cesarz et al. (2019) recommended using a maximum of about 1 cm of soil height during the extraction process. Mobility can be increased significantly when soil aggregates are broken up by gentle sieving at 5 mm or even 2 mm (Cesarz et al. 2019). In the Soil BON Foodweb protocol, we opted for gentle homogenization of the sample by hand to minimize processing time and soil disturbance. Further, when extracting nematodes from dry soil, longer extraction periods (up to one week) are often required as some nematodes are in non‐active anhydrobiosis stages. This can be adapted based on individual researchers' needs, but in Soil BON Foodweb, we opt for a standardized duration of three days at room temperature (20°C–25°C) as we are interested in the active individuals of the nematode communities performing functions (Potapov et al. 2022). Further, extraction ideally should be done from freshly collected samples to minimize storage effects (Ghaderi et al. 2025).

After extraction, nematodes are killed by heat shock, with a sudden rise of water temperature from 20°C to 60°C by holding the rack with vials for 60 s in a pot with almost boiling water or by adding boiling water in a volume ratio of 1:1 to each vial. For long‐term storage and morphological identification, 4% formalin (formaldehyde) or 1–2 drops of TAF [14.9% formalin stock (35%), 1.5% triethanolamine, 83.6% distilled water] is used, but it restricts DNA extraction, which is a goal of Soil BON Foodweb. Therefore, the use of 96% ethanol is recommended instead, notwithstanding the fact that alcohol dehydrates the tissues to such an extent that morphological structures are no longer preserved and make morphological identification impossible. The storage solution (formalin/TAF for morphological identification or ethanol for molecular studies) should be chosen depending on the goals of the individual project.

### 2.1 Critical Steps and Tips

**FIGURE 2:**
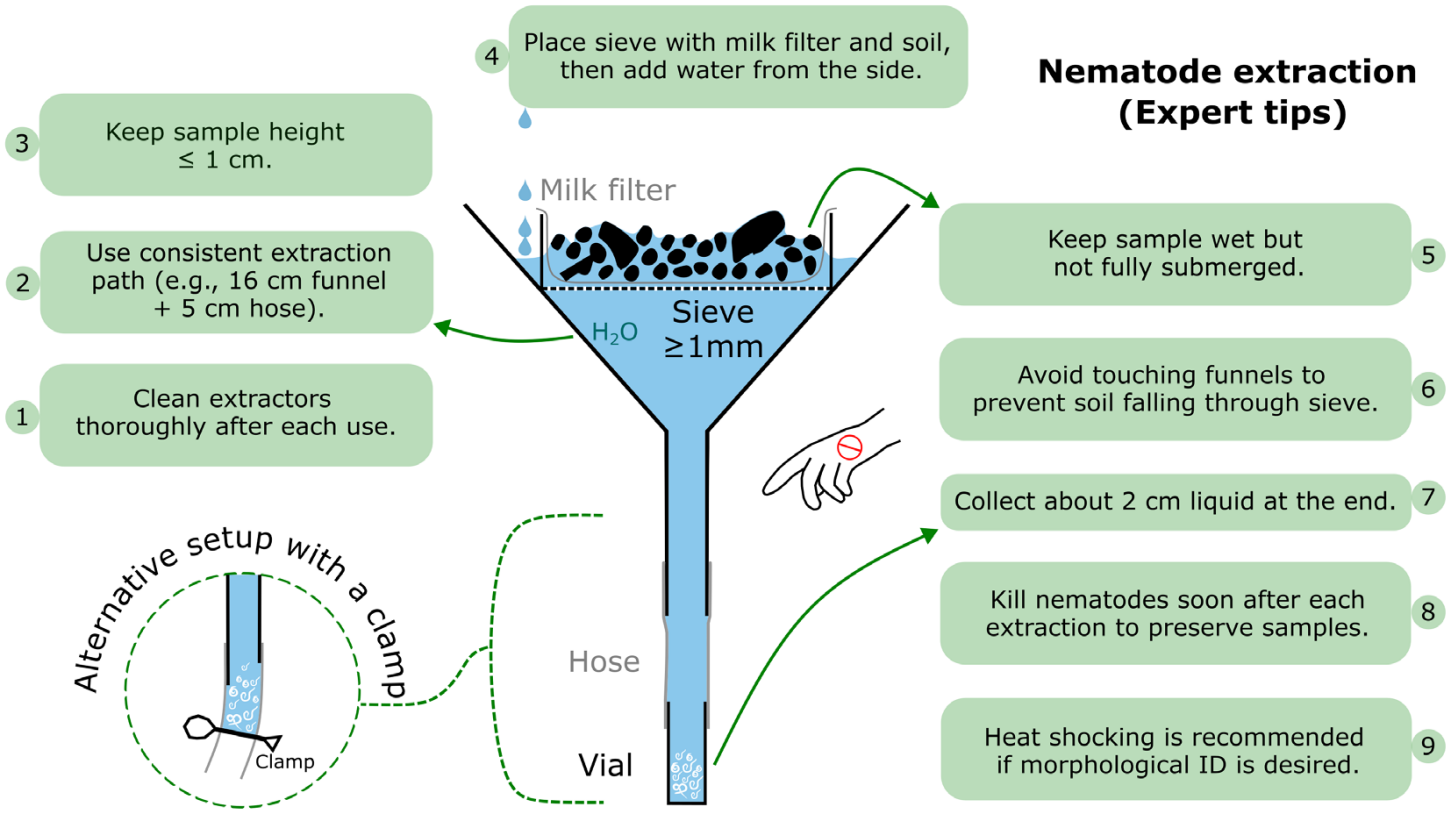
Schematic representation of a Baermann funnel and practical tips from the Soil BON Foodweb expert team for achieving comparable and clean nematode extractions (see video guide on nematode extraction: https://www.youtube.com/watch?v=aKYANMOTBUA).

1. Thoroughly clean all parts of the extractor after each extraction using water to prevent contamination of the samples by soil and animals from previous extractions (Figure 2). This is important because nematodes can tolerate drought by adopting a resistant stage and reviving after rehydration.
2. The extractors in different laboratories should have a comparable length of extraction device (i.e., the distance that animals have to overcome during extraction). We recommend using a kitchen funnel of the total length of 16 cm and 5 cm long connecting hoses (up to c. 20 cm of travel distance).
3. The height of the sample on the sieve (soil thickness) should not exceed 1 cm.
4. Place the sieve with milk filter and soil in the funnel BEFORE you pour in the water. Water should be added slowly from the side between sieve and funnel. Do not wet the soil directly from above, as this will lead to a dirty sample.
5. The sample should touch the water column, but it should neither be soaked nor fully submerged during the extraction to avoid anoxic conditions. Stop adding water as soon as the soil begins to change color or “twitches”, indicating contact with water from below. Wait a few minutes, then check the sample again, adding more water only if the soil surface still appears dry.
6. Do not move or touch the funnels during extraction to prevent soil particles from falling into the nematode sample.
7. Do not let all the water from the funnel run into your sample, and do not rinse the funnel walls, because the lighter soil particles on the funnel walls will then end up in the samples. Collect only the bottom of the extract (volume in vial or if the funnel stem/hose ends in a clamp: roughly 2 cm above the clamp; this also applies to enchytraeid extraction; see Figure 2).
8. It is important to kill the nematodes immediately after the end of the extraction, so that (1) predatory nematodes or other nematode predators (e.g., tardigrades) cannot further injure or consume the others, potentially resulting in drastically reduced nematode counts (Hohberg and Traunspurger 2005).
9. Heat shock killing is recommended so that a flat and typical posture is preserved in nematodes. This is a prerequisite for morphological identification and facilitates body size measurements. This can be done by adding boiling water 1:1 to the extracted nematodes, leading to a sudden rise in temperature to 60°C. Alternatively, if your extractor uses a vial inserted into the extraction hose, you can remove and hold the vial in a water bath (initially 100°C) for one minute to have the same effect.

## 3 Enchytraeidae Extraction

Enchytraeid worms (Annelida, Enchytraeidae) are small whitish relatives of earthworms, ranging from 1 to 30 mm in length and with a body width of less than 2 mm, which places them into mesofauna (0.1–2.0 mm body width; Swift et al. 1979). Due to their soft and non‐sclerotized bodies, enchytraeids must be extracted from intact soil samples (soil cores) to prevent damage during processing. Although several methods can be employed, the most widely used is wet extraction based on migration from heated soil samples through a fine mesh into collection vessels (O'Connor 1955). Alternative methods include (1) flotation with Ludox (colloidal silica) followed by gentle mixing and finally counting the animals when they float at the surface of the solution, (2) dry sieving of leaf litter, and (3) elutriation methods that use the principle that these soil invertebrates are lighter than soil particles and hence sink less rapidly in an upward flow of water.

Flotation with Ludox was suggested by Phillips et al. (1999) as a method superior to the O'Connor method, but specimens die shortly after extraction, and Ludox requires health and safety measures (Adl 2007). This method may be useful for a quick on‐site evaluation of enchytraeid population densities (Phillips et al. 1999) but has never been used in field studies. Dry sieving of leaf litter can yield high enchytraeid numbers, but many small specimens will be overlooked (Healy and Rota 1992). The elutriation method, never used in enchytraeid field studies, involves elaborate procedures that are cumbersome and time‐consuming, and usually requires expensive equipment (Edwards 1991).

The wet extraction has been shown to extract more than 95% of the total number of enchytraeids from organic soils in 3 h and utilizes easy‐to‐get equipment (O'Connor 1955, 1967). Therefore, the wet extraction was the final choice for extracting enchytraeids within the Soil BON Foodweb (Data S2: Wet hot extractors). There are some variations to this basic funnel technique, which diverge in terms of heating temperature (i.e., heat vs. cold extraction), duration of the extraction time, and water level for soaking the sample. Because warming reduces the oxygen concentration of water, oxygen limitation can kill enchytraeids before escaping the soil. To avoid this, a modified wet extraction method was developed in which heating is omitted and the extraction time is extended to several days for organic soils, and up to two weeks for mineral soils (Schauermann 1983; Graefe 1984). In a comparison of extraction methods between several laboratories, the cold method (at room temperature, usually 20°C–25°C) came out as best in terms of efficiency (Didden et al. 1995) and has become the protocol (but with extraction time set to 24 h) used by the International Organization for Standardization (ISO) as ISO 23611‐3 (ISO 2019). However, another comparison of methods (Kobetičová & Schlaghamerský, 2003) did not find statistically significant differences in extraction efficiency. Also, the long extraction duration of the “cold and wet extraction method” can lead to more damaged and dead animals in the sample.

Extraction efficiency can even be higher with heat in some soil types, e.g., in the case of peat soils from British moorlands (35°C surface water temperature over 6 h; Kwon et al. 2000). Therefore, the Soil BON Foodweb Team opted for a combination of 2 h cold extraction followed by 4 h heat extraction (e.g., heat lamp) to reduce processing time when dealing with a large number of samples, while yielding high efficiencies from samples collected in organic‐rich soils (Briones et al. 1997).

### 3.1 Critical Steps and Tips

**FIGURE 3:**
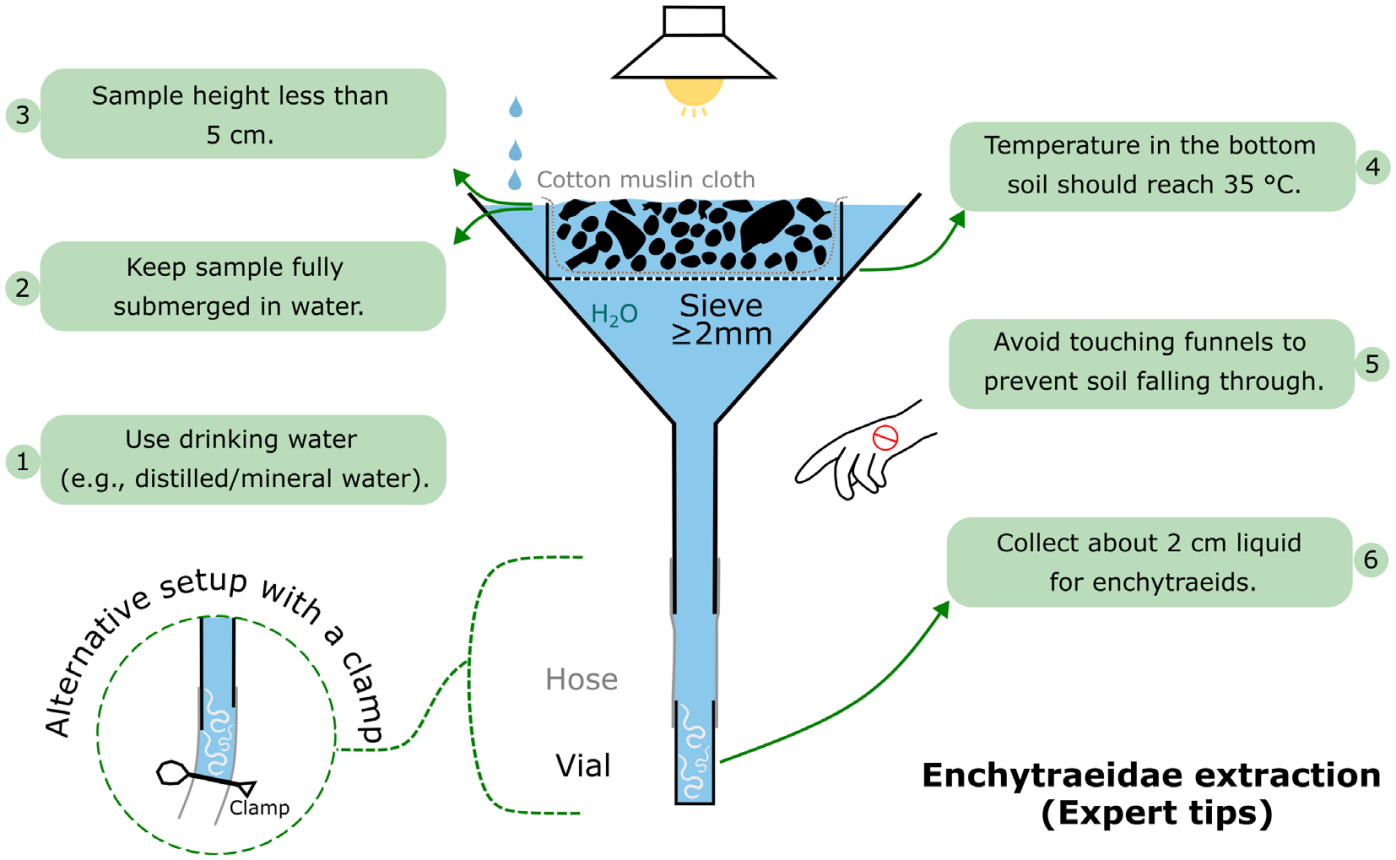
Schematic representation of funnel extractor of enchytraeids and practical tips from the Soil BON Foodweb expert team for achieving comparable and clean extractions.

1. Use deionized or mineral water with low mineral content, if possible. Tap water can also be used, but it should be tested or filtered beforehand: it may contain chlorine, copper, or other compounds toxic to enchytraeids (Figure 3). Chlorine will evaporate if the water is left in open vessels for 2–3 days. Copper, released from the water pipes, can be avoided by letting the water run for some minutes before use. When acid soils are extracted, water pH should be adjusted to 4.5 or 5 with drops of HCl, because some acidophilous species die in pH‐neutral water. Water quality is acceptable when most extracted enchytraeids are complete and alive and stay so (in the fridge) for more than a day.
2. The samples should be fully submerged in water (expert opinion—encourage empirical tests).
3. When soil cores with diameter < 5 cm are used, a greater height (up to 5 cm) can be chosen. Best extraction results are achieved if the sample height does not exceed 2.5 cm. Compact samples may be broken carefully into smaller pieces.
4. Temperature in the upper part of the extractor should reach 40°C; however, the temperature at the bottom of the sieve is critical; it should reach 35°C or more. The temperature at the bottom of the tube, where the worms accumulate, is usually not affected by the heating device above, but cooling is recommended when extraction is carried out in hot weather (ambient temperature 35°C and more).
5. In order to prevent excessive amounts of soil particles falling through the mesh, cover the bottom of the sieve with coarsely woven cotton or muslin cloth before placing the soil sample. In case of uncertainty about the suitable type of cloth, hold it against the light and stretch it; mesh holes of about 1 mm diameter should be seen. Such cloth allows passing of worms while retaining most of the soil particles. However, do not move or touch the funnels during extraction, because loose soil particles may fall through.

6. During extraction, worms assemble at the very bottom of the tube, and releasing the bottom 2–4 cm of water into a Petri dish will collect all the extracted worms, alive and moving in the about half‐filled Petri dish. From there, they can be transferred or photographed. In clayey soils, fine particles can make the extraction water turbid and render high‐resolution photos impossible. Such extracted samples can be left, in Petri dishes, in the fridge (4°C) overnight; clay particles will settle, and the animals can be found alive on the surface of the clay layer. From there, they can be transferred (pipetted) into ~96% ethanol if applicable.

## 4 Microarthropod Extraction

Microarthropods are small arthropods with body widths ranging from 100 μm to 2 mm, included in mesofauna (Swift et al. 1979). Dominant groups of microarthropods are springtails (Collembola) and mites (Acari), but also include proturans (Protura), diplurans (Diplura), small myriapods (Symphyla, Pauropoda), and small larvae of flies (Diptera), butterflies and moths (Lepidoptera), pseudoscorpions (Pseudoscorpiones), and beetles (Coleoptera) (Lavelle and Spain, 2001). Most of the extraction devices for microarthropods are based on their light‐, heat‐, and drought‐escape behavior. An intact soil core (normally in the form of a cylinder or monolith) is placed on a sieve resting inside a funnel, and beneath the end of the funnel stem, a jar or a vial with a fixative solution is placed. A heat source is placed above the funnel to ensure soil drying from the top. Microarthropods start moving down the soil through natural cavities and pores, and fall through the sieve into a jar. The currently used extraction devices are mainly modifications of the Berlese and Tullgren extractors (Berlese 1905; Tullgren 1918). Their extraction efficiency generally varies between 25% and 80% depending on the soil type, light bulb wattage, and thermal regime in the extraction room, as well as extracted taxa (Coleman et al. 2018; Gongalsky 2021; Bruckner 2024). To increase extraction efficiency, several modifications have been proposed. The most important one is cooling of the lower parts of the funnel in addition to heating from above, which creates a marked temperature gradient (MacFadyen 1962). Other modifications include the gradual increase of temperature during extraction (Zaitsev et al. 2002), the use of infrared lamps or electric heating plates, or the use of infrared lamps that illuminate the soil in short pulses and thus minimize mortality of invertebrates from overheating (Kempson et al. 1963).

In addition to dynamic methods for extracting microarthropods from the soil, there is a number of mechanical methods based on the principles of flotation, substrate vibration, and centrifugation (see Krivolutsky et al., 1995 for a review). However, for the Soil BON Foodweb Team, such approaches were not considered, as they are very sensitive to the type of soil investigated, operator‐dependent, as well as resource‐ and time‐consuming. Therefore, the expert team opted for the basic Berlese/Tullgren extraction (Data S3: Dry hot extractors). This method requires the construction of extractors; however, that involves relatively little monetary investment. We also provide schemes and manuals for constructing these devices to make them more accessible (https://zenodo.org/records/18674551).

Although the general extraction principles are straightforward, we urge operators to pay attention to several details. First of all, since most microarthropods cannot efficiently burrow through the soil, and the largest ones typically live on the surface, it is advisable to extract the soil cores upside down and have some free space on the edge. When extracting loosely structured soils, the sample should form a layer of no more than 3–5 cm thick with some space around the substrate for large animals to move down. Second, we recommend using additional layers of mesh (mesh size 1–2 mm) to prevent soil from falling into the collection jars and ensure clean extraction. This is essential for both image analysis and any other subsequent visual processing of the material. Although fine mesh may exclude large microarthropods (Reca and Rapoport 1974), we prioritize sample cleanness in this protocol and note that large invertebrates (> 3 mm body length) are collected by hand sorting (described below). Third, another important point is to make sure that the substrate is completely dry at the end of the extraction because some animals will not leave the soil if moist microsites remain. Therefore, different soil types require different extraction times (typically 3–4 days is needed for extraction from litter and 7–10 days for extraction from soil). It is advisable to check the moisture content of the sample during the extraction by inspecting the bottom of soil samples. In case of intact soil monoliths, it is recommended to assess the moisture at the center of the core after the extraction and extend the process if it remains moist‐to‐the‐touch. Never check the moisture of the sample directly above the extraction device, as this causes a lot of particles to fall and make the extracted sample dirty. Fourth, a gradual increase in temperature helps prevent sample overheating, and a slower extraction process is often more efficient. The surface temperature of the samples should not exceed 50°C. Regularly monitor both the temperature regime and ethanol level in the collection jars. Standardized lab comparisons could help to estimate potential differences in extraction efficiencies among various laboratories caused by operational differences in the procedure (see section *Ring test*).

### 4.1 Critical Steps and Tips

**FIGURE 4:**
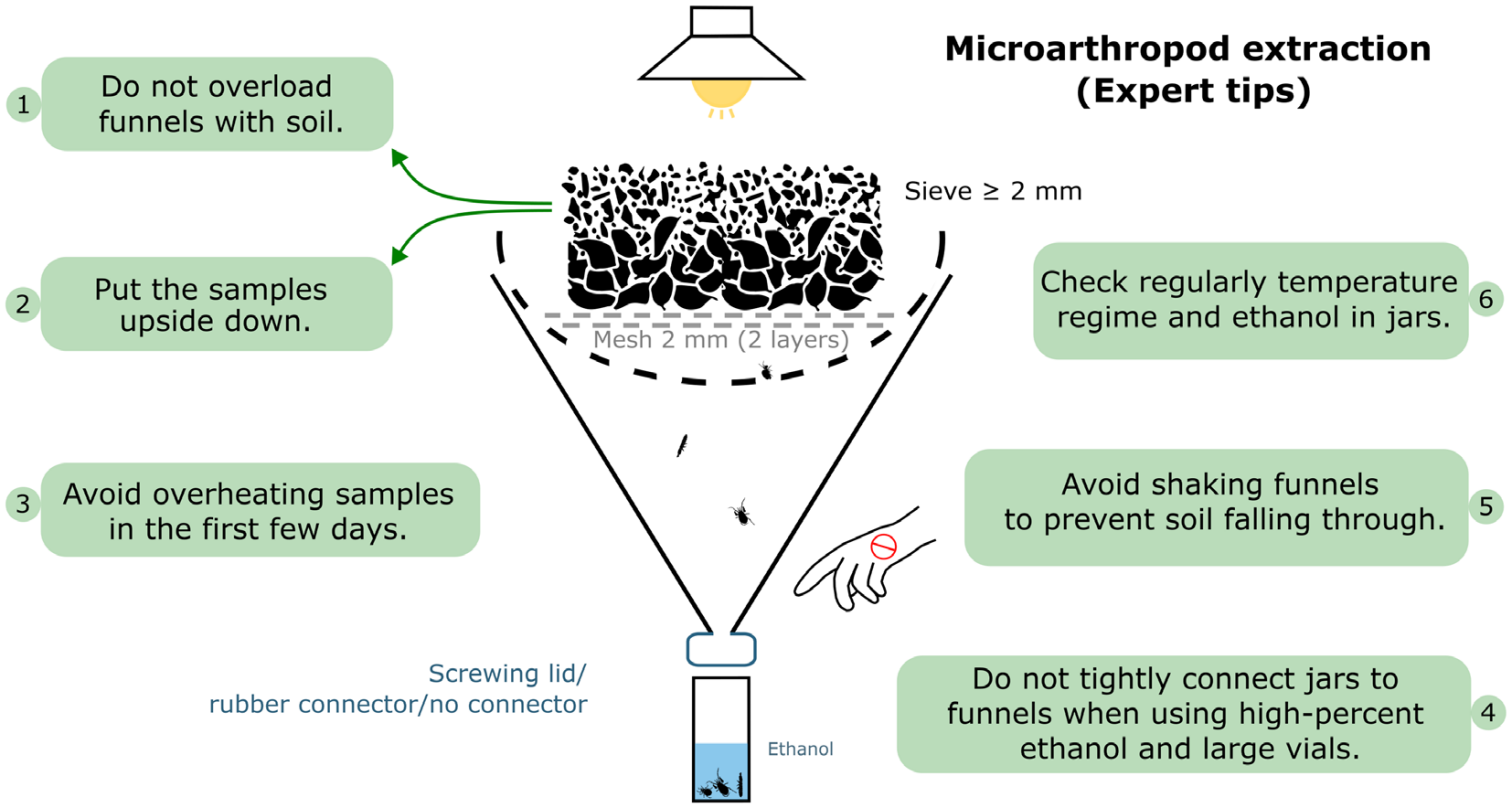
Schematic representation of dry hot extractors and practical tips from Soil BON Foodweb expert team for achieving comparable and clean extractions.

1. Do not overload the funnels with soil. If there is no free space around the edge, or the layer is too thick, animals will not find their way out (Figure 4).
2. Put the soil samples upside down so animals can move through larger pore space, and this also reduces loose soil falling into the jars.
3. Avoid overheating samples in the first few days of the extraction. This can lead to the death of microarthropods. Slow extraction is often more efficient.
4. If possible, extraction jars with wide mouths should not be tightly connected to funnels because intense alcohol vapor from the jars may reduce extraction efficiency (A. Zaitsev, pers. obs.). For extractors, where funnels are tightly connected with tubes, extraction into low percentage ethanol solution (60%–70%; expert opinion—encourage empirical tests) or 50% ethylene glycol, followed by subsequent transferring of extracted animals into high‐percentage ethanol is recommended. Using small‐diameter vials is also beneficial to minimize evaporation.
5. Avoid shaking or any kind of vibration in the room during extraction, for example caused by cleaning activities. When unloading extractors, first carefully remove jars with animals and then the sieves with soil to prevent additional soil particles from falling into the collection jars. Never do any manipulations while soil is in place above the extractors.
6. No matter whether you have a computer‐controlled or a manually controlled extractor, check your samples regularly for the temperature regime (≤ 50°C) and levels of ethanol in the jars.

## 5 Large Invertebrate Extraction

We define large invertebrates as all invertebrates with body width of > 2 mm and body length of > 3 mm. We on purpose do not use the term macrofauna (invertebrates with body width > 2 mm; Swift et al. 1979), because in many studies macrofauna is defined by taxonomic groups and not solely by body size (Gongalsky 2021). Here, we follow body size criteria because large microarthropod groups (e.g., large springtails and mites) cannot be efficiently collected from small soil cores because of their high mobility. On a side note, large ground‐dwelling animals are collected most effectively with Barber pitfall traps—jars dug into the soil with the rim being at the same level as the soil surface (Spence and Niemelä 1994). However, this approach estimates invertebrate activity rather than density (abundance per area), which is one of the criteria in the Soil BON Foodweb Team methods, so we excluded pitfall traps as well as other non‐quantitative collection methods from consideration below.

Quantitative methods for soil large invertebrate assessment are based on the extraction of animals from soil samples of defined area sizes and depths, usually spanning from 10 to 50 cm in diameter or side length, or even larger in the case of litter samples. The oldest and most efficient method is the direct counting of animals in the soil under a microscope without any extraction (André et al. 2002), but this method is extremely laborious and thus rarely used. Probably the most common method is hand sorting (i.e., carefully breaking up the soil and manually collecting the animals) of litter and soil samples, either in the field or in the laboratory (Gilyarov 1941). This method is laborious but can be applied with minimum equipment (a spade and a plastic tray) and requires little training. Hand sorting is the standard method in the Tropical Soil Biology and Fertility (TSBF) protocol (Anderson and Ingram 1993; Römbke et al. 2006), and produced valuable data summarized in the recent global databases of soil macrofauna (Lavelle et al. 2022; Mathieu et al. 2022). The time that can be saved and the resultant reductions in extraction efficiencies when sorting is time‐limited have been quantified for earthworms (Schmidt 2001a). However, to give universal recommendations on time‐limited sorting, a test covering diverse soils and settings across laboratories following the same protocol would be required.

Heat extraction (see above) can also be used for extraction of large invertebrates from litter and soil samples, but this requires large equipment and may lead to a different representation of taxa compared to hand sorting (see the results from our *Pilot* Experiment 2). Published comparisons suggest that heat extraction underestimates the abundance of earthworms (in particular endogeic and anecic earthworms are undersampled) and social insects (ants and termites), while hand sorting underestimates the abundance of small taxa, e.g., Thysanoptera (Gongalsky 2021). As a variation of heat extraction, the Winkler extractor is often applied in the tropics to extract invertebrates from litter. This extractor consists of a coarse‐mesh bag with substrate, which is placed inside a cloth container suspended over a collection jar at the bottom (Sabu et al. 2011). However, social insects are often poorly recovered by Winkler extractors as well, so alternative methods such as counting the number of nests at large spatial scales, e.g., per hectare (Delabie et al. 2021), have been proposed. Additionally, a comprehensive assessment of earthworm communities may require, in some cases, sampling deep soil down to 1 m. As alternative approaches (or in combination with hand sorting), dynamic extraction of earthworms is possible, in situ, using mustard oil (allyl isothiocyanate) or electrical devices (Schmidt 2001b; Čoja et al. 2008).

In the expert team, we opted for hand sorting of a quadrat of 25 × 25 cm (the standard TSBF area) and 10 cm depth (a compromise solution in comparison to 20 cm in TSBF to reduce labor) to extract large invertebrates. Our main motivation was feasibility of this method under diverse climatic conditions and soil types due to low requirements of extraction equipment and usually low transportation costs. In addition, hand sorting ensures better comparability of collected data with the existing global‐scale macrofauna initiative (Mathieu et al. 2022). Although it can underestimate some smaller or deep‐burrowing taxa, it was proven to render better results than chemical extraction for earthworms (Briones and Schmidt 2017). Moreover, hand sorting also provides good estimates of less mobile animals like gastropods and some insect larvae, which might be underestimated by heat extraction. Although we recommend hand sorting to take a minimum time of 45 min per sampling point (two people) and until the entire sample is thoroughly checked, the method is still very prone to human biases because every observer will have different diligence and detection abilities. To assess the accuracy of hand sorting, we conducted pilot Experiment 2 comparing the number of heat‐extracted animals from the same soil samples that were hand‐sorted prior to the heat extraction. Our aim was to see which taxa are usually omitted using hand sorting (see section *Pilot Experiment 2*).

### 5.1 Critical Steps and Tips

**FIGURE 5:**
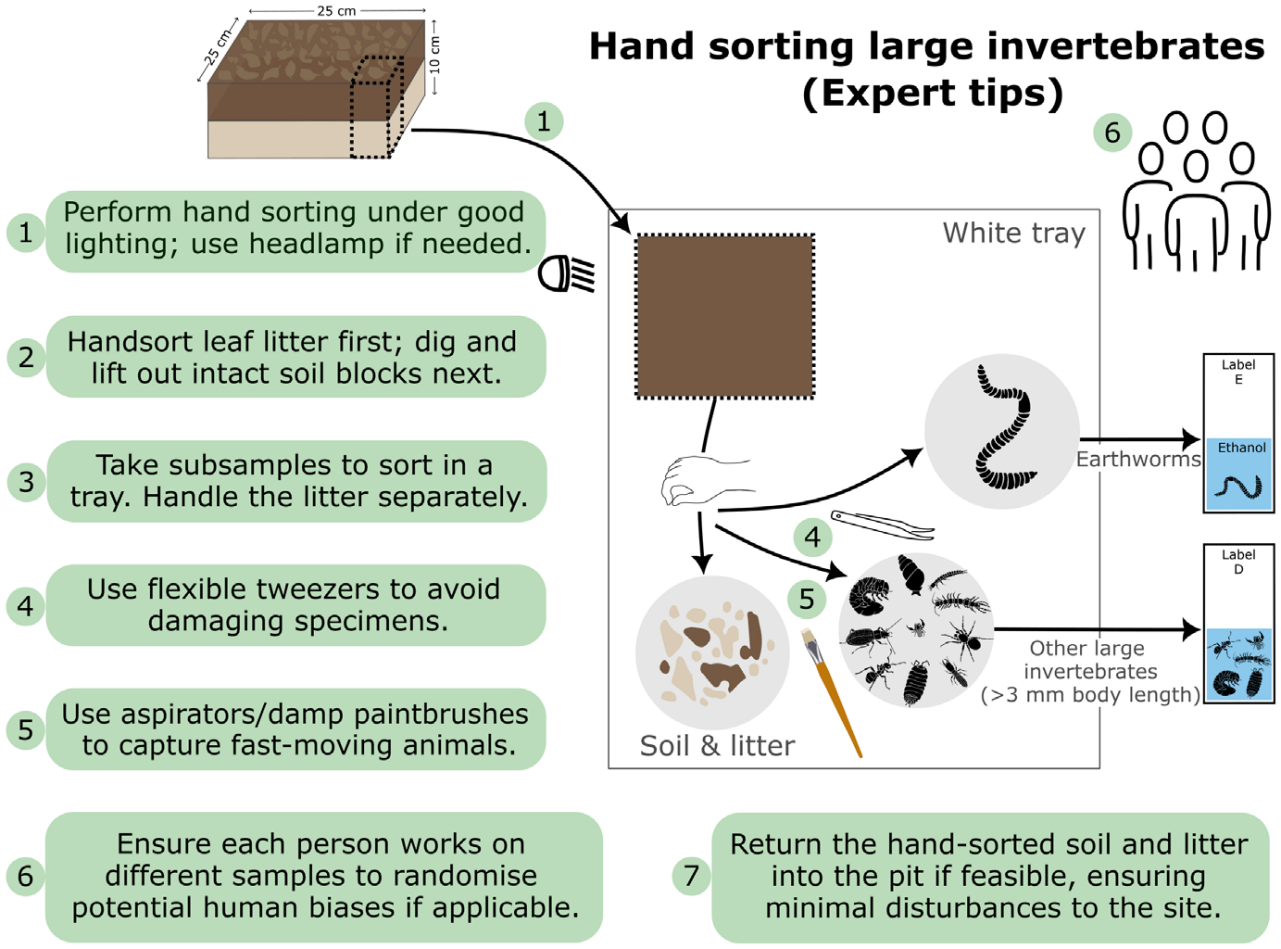
Schematic representation of hand sorting of earthworms and other large soil invertebrates, and practical tips from the Soil BON Foodweb expert team for achieving comparable extractions.

1. Hand‐sort leaf litter layers initially. Then, dig quickly to reduce animals escaping. Cut straight and clean along the edges, lift out intact soil blocks. Do not cut inside the block to reduce damage to animals.
2. Hand sorting should be done under good ergonomic and light conditions. When hand sorting is done in the field, use headlights in case of shady conditions (Figure 5). Use a white background (Point 3) to detect animals easily.
3. Use a large plastic sheet or box, take small subsamples to break them up and check them, and move soil from unsorted to sorted and from one side to the other in a systematic fashion. Carefully inspect plant roots, twigs, folded leaf litter, and the content of decaying fruits, acorns, nuts, etc.
4. Use flexible (soft) tweezers that do not damage specimens during collection.
5. To catch small, quickly moving animals use entomological aspirators, paintbrushes, or put a drop of ethanol on an individual before transfer.
6. When multiple people are performing the hand sorting, randomize people's allocation and have each sample processed by more than one person to evenly distribute individual variation across treatments.
7. Return the hand‐sorted soil into the pit if feasible, ensuring minimal disturbance to the site and reducing subsequent risk of injury to animals and people.

## 6 Pilot Experiment 1: Extraction Efficiency of Nematodes Using One vs. Two Milk Filters

The cleanliness of the samples is crucial to developing automated recognition, counting, and biomass estimation using supervised machine learning (Sys et al. 2022). Overlaps between animals and soil particles could blur the shape of the individuals and challenge their detectability by machine learning algorithms. Therefore, we tested whether the use of two milk filters in the nematode extraction could provide cleaner samples and whether it could influence extraction efficiency compared to the use of only one milk filter.

The extractions were performed in two separate laboratories (Finland—University of Jyväskylä and the Czech Republic—Biology Centre of the Czech Academy of Sciences). In Finland, samples were taken from two boreal coniferous forests on sandy soils, one Scots pine dominated and another Norway spruce‐dominated stand, while in the Czech Republic, samples were taken from a spruce forest on loamy soils. At each of three localities, we sampled 5 soil cores (5 cm inner diameter) to a depth of 10 cm. At the laboratories, all samples were first carefully mixed by hand, and then two subsamples were taken for extractions (12–21 g fresh weight). The small sample size may underestimate certain nematode groups (Ghaderi et al. 2025), but for testing effects of filter number, it should not be a concern for this experiment. One subsample was extracted using one milk filter and the other using two milk filters. The extraction procedure followed the Soil BON Foodweb Team protocol (Data S1: Wet cold extractors). Extracted nematodes were counted under a microscope. To evaluate differences in the numbers of nematodes extracted with one or two milk filters, we used a two‐way ANOVA in R v4.5 with location and number of filters as independent factors.

**FIGURE 6:**
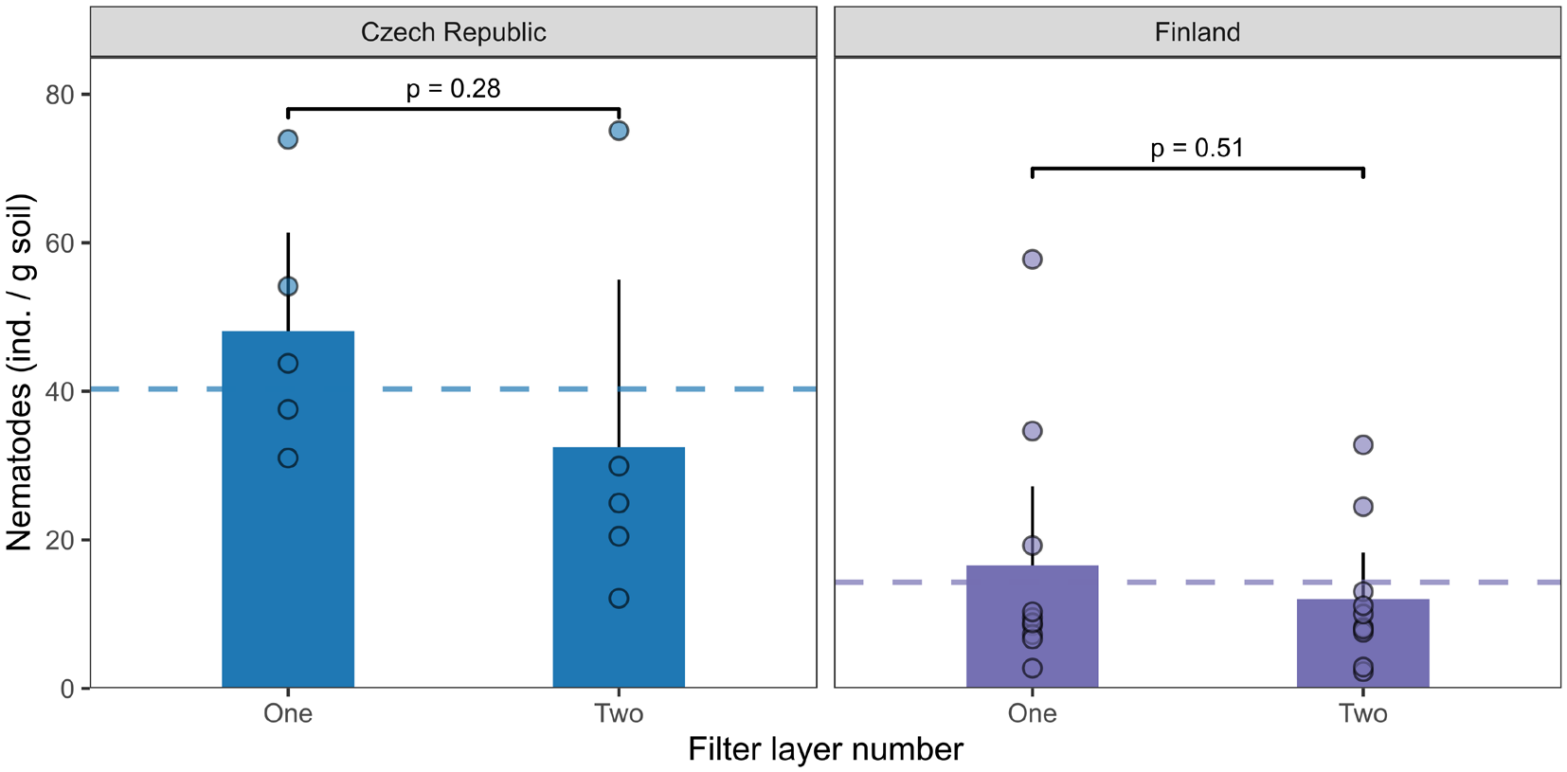
Mean number of nematodes extracted from 1 g of fresh forest soil sample using either one or two milk filters at two laboratories (Czech Republic and Finland). Bars represent means, and error bars indicate upper 95% confidence intervals. The dashed line shows the mean for each country.

We found no difference between the numbers of nematodes extracted with one or two milk filters (Figure 6; F_1,25_ = 1.88, *p* = 0.18). Against our expectations, clean samples were obtained in both one‐ and two‐milk filter extractions. We speculate that the sample cleanliness probably depends more on the other factors, such as soil treatment during extraction, soil clay content, initial soil moisture, milk filter type, and number of animals in the sample. It has been previously shown that the filter type is crucial for extraction efficiency and may differ considerably depending on the used material and soil type (Cesarz et al. 2019). Although the milk filters used in two studied laboratories were from different suppliers, similar treatment effects were observed. Overall, although their effects on community composition and sample cleanliness are to be evaluated in future studies, our results suggest that two layers of milk filters do not significantly reduce the abundance of extracted nematodes, and this approach may be tested in cases where samples are particularly ’dirty’. A ring test could be an optimal solution to ensure data comparability (see section *Ring test*).

## 7 Pilot Experiment 2: The Efficiency of Hand Sorting for Large Soil Invertebrates

To test the efficiency of the hand sorting method, we first hand‐sorted litter and soil samples and then subjected the same, already sorted soil and litter to Kempson's dry and heat extraction. To evaluate differences among teams working in different regions, we performed this test in three independent laboratories: the Czech Republic (CZE, Biology Centre of the Czech Academy of Sciences), Germany (GE, University of Leipzig), and Italy (IT, Eurac Research). In the Czech Republic, samples were taken from two mixed spruce‐ and beech‐dominated forests (USDA soil texture class: loam). In Germany, samples were taken from two forests (beech‐ and oak‐dominated, silty loam) and two meadows (loam to silty clay loam). In Italy, the samples were taken from one oak (sandy loam), two subalpine spruce, and two subalpine larch forests (loam). The soil samples were obtained following the Soil BON Foodweb protocol (Potapov et al. 2022) by digging out a soil monolith of 25 × 25 cm square of soil, including the litter layer and the underlying 0–10 cm of soil. The vegetation was cut and removed before digging. The litter was collected and hand‐sorted separately from the soil. The samples were transported to the laboratory and hand‐sorted to catch all large invertebrates (> 3 mm in body length) except enchytraeids. The screened soil and litter samples were then put separately in Kempson's extractors (Kempson et al. 1963) and extracted for five days (or until the sample was completely dry) in order to expel all the remaining animals from the soil. In Germany, a 25% subsample of each sample was used for heat extraction due to limited space in the extractors. Extracted invertebrates were stored in pure 96% ethanol at −20°C and later identified to high‐rank taxa (orders, families) under a dissecting microscope. The percentage of missed individuals for each taxon was calculated as the number of Kempson‐extracted individuals divided by the total number of individuals collected (hand sorting + Kempson). To compare between laboratories and animal groups, the proportion of missed individuals was evaluated by linear‐mixed effect models using R v4.5, with laboratory, animal group, and their interactions as fixed factors while plot ID was included as a random factor.

**FIGURE 7:**
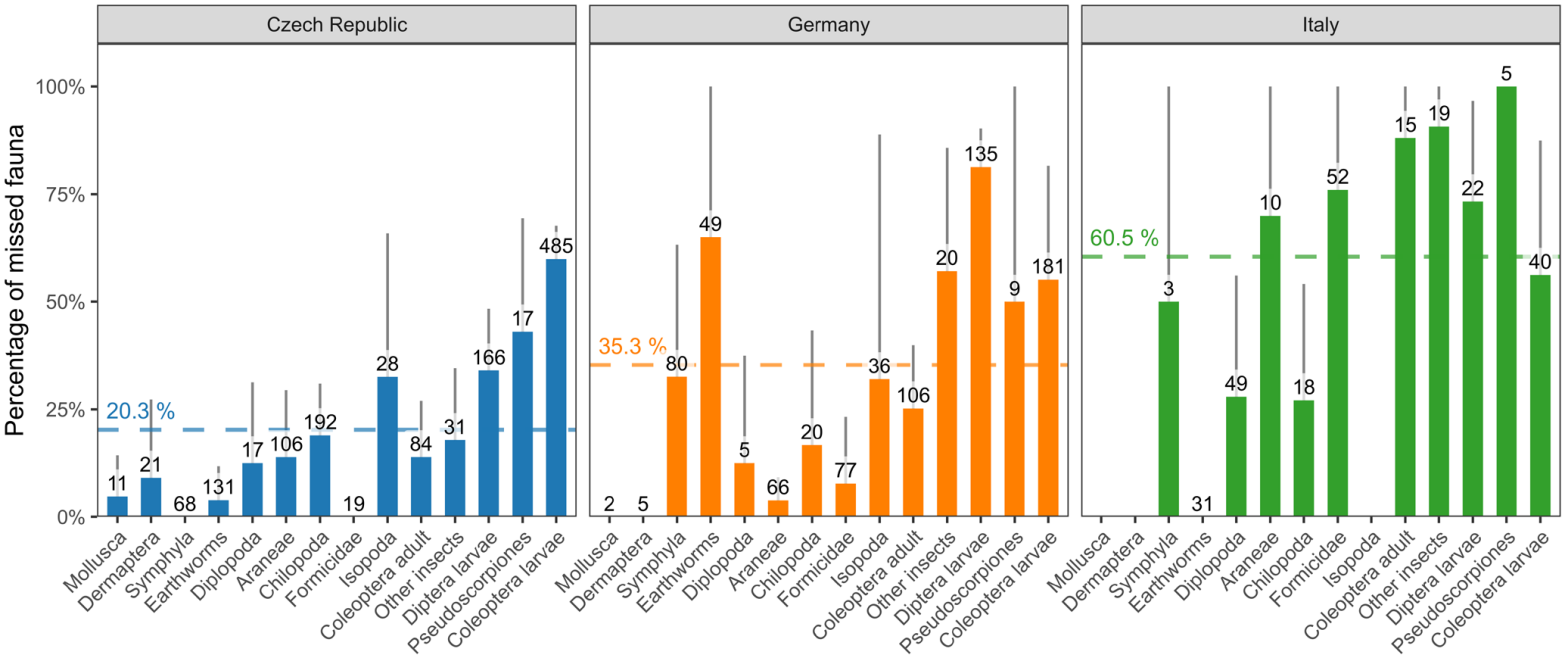
The average percentage of missed soil fauna individuals during hand sorting of soil monoliths in three laboratories (the Czech Republic, *n* = 20; Germany, *n* = 8; Italy, *n* = 10). The percentage is the number of individuals extracted with Kempson's apparatus divided by the total number of (hand‐collected and extracted) individuals across all samples in a given laboratory. These total numbers are shown above the columns. The dashed line represents the average percentage of missed individuals across all taxa in each laboratory. The category ‘Other insects’ contains all insect groups (e.g., Hemiptera and Lepidoptera) not belonging to the dominant groups of soil fauna. Earthworms are Lumbricina.

In general, our results show that there are overlooked individuals in virtually all soil animal taxa. On average across taxa, 31.3% of individuals were missed, but the percentage of missed individuals varied strongly depending on the taxon (taxon effect F_13,276_ = 6.5, *p* < 0.001). The most missed animal taxa across all three laboratories were Pseudoscorpiones (64.4% ± 46% missed on average across three laboratories), Diptera larvae (62.9% ± 35%), Coleoptera larvae (57.1% ± 33%), and other insects (55.2% ± 45%). Coleoptera and Diptera larvae were also the most abundant taxa across all samples. Although overlooked in some cases, the overall missed percentages were low for Araneae (29.3% ± 36%) and Lumbricina (23.0% ± 33%; Figure 7). Individual laboratories also differed in the collecting efficiency of specific taxa (taxon: laboratory interaction F_23,276_ = 3.7, *p* < 0.001). Based on ANOVA of each animal group, significant differences were observed between laboratories in Lumbricina (CZE = 3.9%, GE = 65.0%, IT = 0%), Diptera larvae (CZE = 34.0%, GE = 81.3%, IT = 73.3%) other insects (e.g., Homoptera, Lepidoptera, CZE 17.9%, GE = 57.1%, IT = 90.7%), Araneae (CZE = 13.9% GE =3.9%, IT = 70.0%), Formicidae (CZE = 0%, GE = 7.8%, IT = 76.0%), adult Coleoptera (CZE = 13.9%, GE = 25.2%, IT = 88.1%) and Symphyla (CZE = 0%, GE = 32.6%, IT = 50.0%). On average across target animal taxa, 20.3% of all individuals were missed in Czech sites, 35.3% in German sites, and 60.5% in Italian sites. Overall, our test showed that efficiency of hand sorting depends on both individual taxa and the collecting team/ecosystem type (it was not possible to separate the latter two factors with our sampling design). Furthermore, individual taxa were overlooked to a variable extent depending on the collecting team, which may likely be explained by differences in the local fauna (e.g., more obscure life forms of Araneae in one location than in another).

The results highlighted two main disadvantages of the hand sorting method from a soil monolith. First, the method misses a significant proportion of large invertebrates (18%–64%), either due to human bias or different local characteristics of the soil and fauna. Therefore, total densities evaluated by hand sorting are likely to be significantly underestimated. Moreover, the number of missing individuals differs significantly among animal taxa, and thus hand sorting skews the information about community structure. The overlooked individuals were especially from the ecologically important and abundant groups of macrofauna, such as the larval stages of Coleoptera and Diptera (> 3 mm). The underestimation might be less pronounced when the animal biomass is calculated, as omitted specimens within taxonomic groups were mainly small in size (visual observations). We speculate that larvae and isopods were missed due to often dense cover by soil particles that makes them hard to recognize. In addition, earthworms were often covered by soil during the sorting, and thus probably obscured and overlooked in the German sites. Moreover, in many of these taxa, thanatosis (immobility in face of danger) could be a protective behavior that could be provoked by hand sorting (Humphreys and Ruxton 2018). For example, ants, typically exhibiting active movement, were less likely to be missed. Overall, the hand sorting method captured actively moving taxa, such as spiders, adult beetles, centipedes, and ants, better. A thorough assessment and large time investment (at least 60 min for one person per 25 × 25 × 10 cm monolith) were needed to collect most animals with this method, and even then, small individuals were overlooked. This critical information needs to be taken into account when interpreting the results of hand sorting.

The second disadvantage lies in the significant differences observed in results coming from different laboratories. One possible explanation could be variable searching time per sample among laboratories. The searching time through one sample was on average, one hour for the four people involved in the Czech team (4 h/sample of soil+litter), one hour for two people from the German team (2 h/sample), and 25 min for 3 people in the case of the Italian team (1 h 15 min/sample). This is aligned with the overall collection efficiency that descends in the order from Czech to Germany to Italy (see above). Given the association between search time and recovery efficiency, we encourage laboratories to first benchmark their own hand‐sorting efficiency against a fixed‐time standard before large‐scale deployment or report the searching time in the methods. However, the searching time cannot be unified to standardize the method because effectiveness is strongly related to the soil type, moisture of the soil, weather conditions, amount of roots, aggregation of soil, and type of substrate (e.g., litter vs. soil), and the number of specimens present. A ring test following our pilot experiment, involving more laboratories and improved methodology, would be necessary to identify main sources of bias and potentially develop calibration coefficients for different animal taxa, ecosystems, and searching time.

## 8 Ring Test

Ring tests involve conducting identical research interventions in geographically distant scientific units following the same protocol to standardize these protocols and ensure comparable results across the units. This approach is commonly used in soil ecotoxicology to draft and quality‐check international standards for research methods and facilities (see e.g., Knacker et al. 2004; Koolhaas et al. 2004; Moser and Römbke 2009). In soil ecology, this approach has to‐date received little attention (but see Crossley and Blair 1991). Results of Pilot experiment 2 presented in this paper show that even with the same protocol, different laboratories achieve variable efficiency of hand sorting. Hence, it is of vital importance to verify soil fauna extraction efficiency in global studies assessing soil communities and in large‐scale monitoring surveys. There are two possible ways to implement the ring test in the global assessment of soil animal biomass, diversity, and functioning. The first and more complex method is to extract artificial communities formed by focal taxa that are either pre‐extracted or taken from laboratory cultures. The second approach is based on extraction of samples from a single natural community in different laboratories. The first option provides detailed taxon‐specific information, valuable due to potential extraction efficiency variation by taxon and functional group. The second approach avoids soil animal stress due to the pre‐extraction. However, there are three major constraints of the second approach. First, many more samples need to be extracted to account for local spatial variability in species distribution. Second, there are numerous legal hurdles to authorizing the shipment of non‐sterilized soil across customs borders. And last, logistical arrangements must be good enough to prevent community changes caused by varying shipment duration and conditions. Still, the second option of ring test implementation might be the preferred one if performed regionally. Globally, we propose two levels of ring testing: (i) standardization and validation of soil animal extraction methods and apparatus in a few core facilities across different regions and then (ii) regional‐level implementation of these standardized methods, including training of participating teams, using samples collected within the same country or customs‐free areas. A pre‐defined pass criterion (e.g., > 80% recovery) for any given laboratory's extraction setup could be considered valid. In the case of studies covering wide geographic ranges, ring tests represent a very promising yet technically and organizationally challenging opportunity to increase the quality, intercomparability, and reliability of the collected data. With this paper, we call for including such ring tests in the agenda of existing and future large‐scale projects and initiatives, and also during the establishment of new laboratories.

## 9 Future Perspectives

Comparable data from different locations is essential to test major (macro)ecological hypotheses related to soil fauna (Mathieu et al. 2022), assess changes of soil communities along wide climatic and disturbance gradients, correctly estimate contribution of soil animals in the global biomass stocks (Bar‐On et al. 2018), and understand global trends of soil biodiversity (Guerra, Bardgett, et al. 2021). Adequate biomass estimates are necessary to implement soil food web and energy flux analyses, to describe variation in functioning of soil animal communities (Jochum et al. 2021; Potapov et al. 2022). Here, we describe the methodological basis for the global‐scale monitoring of soil animal communities in the framework of the Soil BON Foodweb. The methodology has been implemented across 30+ countries globally in 2022–2023, and will continue in 2025–2026, 2028–2029, and beyond to provide realistic assessments on the status, trends, and drivers of soil animal biomass and community composition (Potapov et al. 2022). Although none of the methods and protocols are perfect to assess soil fauna comprehensively, the Soil BON Foodweb expert team has put a great collaborative effort into finding balance between labor, financial investments, and representativeness of each method. Despite the fact that these methods described above are not new and are used routinely in many laboratories, many users report inconsistencies in results. By summarizing ‘expert tips’ and providing visual materials, we aim at fostering better standardization and practical implementation of transnational soil biodiversity assessments. Furthermore, we call for a global‐scale ring testing of extraction methods of soil fauna as the next crucial step towards understanding the global soil animal diversity status and trends. Similar standardization has been implemented for the assessment of other biodiversity dimensions like soil microorganisms and soil microbial functions (e.g., in Soil BON; Guerra et al., 2021) via molecular techniques and centralized laboratory facilities. However, this is not possible for most soil fauna taxa, and we advocate for the establishment of local facilities and building of local expert capacity.

Our recommendations are intended to reach scientists beyond the Soil BON Foodweb. We invite soil ecology laboratories to join our efforts in the global standardization of soil animal assessment and monitoring by making small amendments to existing protocols or expanding their toolbox. Our protocols can be used in local, national, or regional assessments and monitoring schemes, ultimately to contribute to the accumulating body of knowledge on soil fauna. In the UN Decade on Restoration and with important initiatives like the EU soil strategy for 2030, including the recently adopted European Soil Monitoring and Resilience Directive being implemented, it is of utmost importance to understand the status and trends in soil biodiversity. For this, time series analyses need to be implemented (Eisenhauer et al. 2022). Many countries are establishing their national biodiversity monitoring programs across taxa. To aid full inclusion of soil biodiversity, standard, open, and cost‐efficient methods need to be agreed upon. Our protocols may serve as a basic (and potentially expandable) reference making such assessments comparable across borders.

## Author Contributions

**Michala Tůmová:** conceptualization (lead), data curation (lead), formal analysis (supporting), investigation (equal), methodology (equal), project administration (equal), resources (equal), validation (equal), writing – original draft (equal), writing – review and editing (equal). **Jing‐Zhong Lu:** data curation (equal), formal analysis (lead), methodology (equal), project administration (equal), validation (equal), visualization (lead), writing – review and editing (equal). **Maria J. I. Briones:** conceptualization (equal), methodology (equal), project administration (equal), supervision (equal), validation (equal), writing – review and editing (equal). **Simone Cesarz:** methodology (equal), project administration (supporting), resources (supporting), supervision (supporting), validation (equal), writing – review and editing (equal). **Nico Eisenhauer:** conceptualization (equal), investigation (supporting), methodology (equal), project administration (supporting), resources (supporting), supervision (equal), validation (supporting), writing – review and editing (equal). **Saori Fujii:** investigation (supporting), methodology (equal), project administration (supporting), resources (supporting), supervision (supporting), validation (equal), writing – review and editing (equal). **Konstantin B. Gongalsky:** conceptualization (supporting), methodology (equal), project administration (supporting), resources (supporting), supervision (equal), validation (supporting), writing – review and editing (equal). **Jari Haimi:** conceptualization (equal), data curation (equal), formal analysis (supporting), investigation (equal), methodology (equal), project administration (supporting), resources (supporting), supervision (equal), validation (equal), visualization (supporting), writing – original draft (supporting), writing – review and editing (equal). **Karin Hohberg:** methodology (equal), resources (supporting), supervision (equal), validation (equal), writing – original draft (supporting), writing – review and editing (equal). **Stephan Jänsch:** investigation (supporting), methodology (supporting), resources (supporting), validation (equal), writing – review and editing (equal). **Hannah Karuri:** conceptualization (equal), methodology (supporting), project administration (equal), supervision (supporting), validation (supporting), writing – review and editing (supporting). **Yudai Kitagami:** conceptualization (equal), investigation (supporting), methodology (equal), project administration (supporting), supervision (supporting), validation (supporting), writing – review and editing (supporting). **Daniil I. Korobushkin:** investigation (supporting), methodology (equal), resources (supporting), visualization (equal). **Alfred Lochner:** conceptualization (supporting), data curation (supporting), investigation (equal), methodology (supporting), project administration (supporting), resources (equal), writing – review and editing (supporting). **Jérome Mathieu:** conceptualization (supporting), methodology (supporting), supervision (supporting), writing – review and editing (supporting). **Oksana L. Rozanova:** investigation (supporting), methodology (equal), resources (supporting), visualization (equal). **Jörg Römbke:** methodology (equal), project administration (supporting), supervision (supporting), validation (supporting), writing – review and editing (supporting). **Julia Seeber:** conceptualization (equal), investigation (lead), methodology (lead), project administration (supporting), supervision (equal), validation (equal), writing – review and editing (equal). **Rüdiger Schmelz:** conceptualization (equal), investigation (equal), methodology (lead), resources (equal), supervision (equal), validation (lead), writing – review and editing (equal). **Olaf Schmidt:** conceptualization (equal), investigation (supporting), methodology (lead), project administration (supporting), resources (supporting), supervision (equal), validation (equal), writing – review and editing (lead). **Clement Schneider:** data curation (supporting), investigation (supporting), methodology (equal), resources (equal), writing – review and editing (supporting). **Xin Sun:** conceptualization (equal), funding acquisition (supporting), methodology (supporting), project administration (supporting), resources (supporting), supervision (equal), validation (supporting), writing – review and editing (supporting). **Jiri Tuma:** conceptualization (equal), data curation (equal), formal analysis (lead), investigation (lead), methodology (equal), project administration (equal), resources (equal), validation (equal), visualization (equal), writing – original draft (supporting), writing – review and editing (equal). **Andrey S. Zaitsev:** conceptualization (supporting), methodology (equal), project administration (supporting), supervision (supporting), validation (equal), writing – review and editing (equal). **Andrey G. Zuev:** data curation (equal), investigation (equal), methodology (supporting), project administration (equal), validation (supporting), visualization (supporting), writing – review and editing (equal). **Anton M. Potapov:** conceptualization (equal), formal analysis (supporting), funding acquisition (lead), investigation (equal), methodology (equal), project administration (lead), resources (equal), supervision (lead), validation (equal), visualization (supporting), writing – original draft (supporting), writing – review and editing (lead).

## Funding

This work was supported by Deutsche Forschungsgemeinschaft, Ei 862/29‐1, FZT 118, 202548816. European Research Council, 101170898.

## Conflicts of Interest

The authors declare no conflicts of interest.

## Supporting information


**Data S1:** Wet cold extractors.

**Data S2:** Wet hot extractors.

**Data S3:** Dry hot extractors.

## Data Availability

Supporting manuals and protocols have been deposited in Zenodo including those from the supporting information: https://doi.org/10.5281/zenodo.18674551
