# Supplementary material for: Standardized Protocols for Soil Fauna Extraction and a Call for Cross‐Lab Implementation
Source: Ecol Evol. 2026 Apr 21;16(4):e73407. doi: 10.1002/ece3.73407 (PMC13099171; doi:10.1002/ece3.73407)
Supplement: Supplementary file 1 — Data S1: Wet cold extractors. [file ECE3-16-e73407-s001.pdf]

Wet cold extractors: Nematode extraction v1.8

Nematode extraction is not mandatory and is done in the Soil BON sampling. [SBF relevant]

VIDEO: https://www.youtube.com/watch?v=8Ennbh0KkUQ

A Baermann funnel extractor is used as the standard. A soil core including litter and 10 cm soil should be divided into two vertical halves (loose samples can be divided by weight). One half is used for nematode extraction, the other for enchytraeid extraction using different extraction methods (wet cold vs wet hot, gently homogenized soil vs intact soil cores, ~72 h vs 6 h extraction time). [SBF relevant] For nematodes:

Materials:

- Milk filters diameter 16-25 cm, 1 per sample
- Vials to store worms (2 ml glass/plastic tubes*), 1 per sample
- Ethanol ~96%, 2 ml per sample
- Printed ethanol-resistant labels, 1-2 per sample
- Drinking water, flushing bottle
- Scales, scissors, knife, spoon, tweezers

Instructions:

1. Put the soil sample (half core) [SBF relevant] for the extraction of nematodes in a plastic bag.
2. Gently homogenize the substrate by hand to destroy large aggregates. Do not smash the soil. Clay and wet soils should be homogenized very cautiously.
3. Weight the sample (half core) [SBF relevant] for nematode extraction and annotate (for expressing the results per g of soil).
4. Take only a subsample of soil and litter that will form a max 1 cm layer on the sieve.
5. Weight this subsample for nematode extraction and write down (for recalculation).
6. Put the milk filter into the sieves before placing them on the funnels.
7. Gently put the weighted subsample on top of the filter (max 1 cm in height).
8. Place the funnels in the funnel holders, attach the vials at the bottom of the hoses.
9. Fill the funnels with drinking water by about a half**. Check if the plugs are closed tightly. Check and remove air bubbles by squeezing the hoses.
10. Gently place the sieves with soil and litter on top of the funnels. The substrate should be fully soaked but not submerged completely! Add more water, if needed.
11. Run the extraction for 72 h at room temperature but remove extracted animals regularly every 24 h to prevent decay and predation (3 times in total).
12. To remove the extracted animals, strangulate the hose with tweezers or your fingers, gently remove the vial, and replace it with the new one filled with water.
13. Terminate nematodes by brief heat shock***. Let the vial with animals settle for 30 min****, and then remove ¾ of water from the top of the vial (nematodes are concentrated at the bottom). Store animals by filling the vial with 96% ethanol [SBF relevant]. Label the vial and store it in a fridge until step 15.
14. When the extraction is finished, gather the last portion of extracted animals, remove the sieve and the water from the funnel and clean the extractor.

15. Put together the three portions of nematodes from the same soil sample collected during the three days: Carefully remove ¾ of ethanol from the top of each vial and transfer the rest of the ethanol with nematodes to one of the three vials.
16. Put the label in the vial and fill it with 96% ethanol. Store the vials in a freezer. [SBF relevant]

**Vials of other volumes can be used. Adjust the amount of ethanol and storage space.*

***Supermarket water is preferred. Tap water should be used after 5 minutes of running and 24h for evaporation. The sieve should not be metal, except it is stainless steel.*

****Heating the nematodes at 60°C induces species-specific posture useful for morphological identification. This can be done by adding boiling water 1:1 to the extracted nematodes, leading to a sudden rise in temperature to 60°C. Alternatively, if your extractor uses a vial inserted into the extraction hose, you can remove and hold the vial in a water bath (initially 100°C) for one minute to have the same effect.*

*****Smaller nematodes are very light and may suspend for up to an hour.*
