# Supplementary material for: Standardized Protocols for Soil Fauna Extraction and a Call for Cross‐Lab Implementation
Source: Ecol Evol. 2026 Apr 21;16(4):e73407. doi: 10.1002/ece3.73407 (PMC13099171; doi:10.1002/ece3.73407)
Supplement: Supplementary file 2 — Data S2: Wet hot extractors. [file ECE3-16-e73407-s002.pdf]

Wet hot extractors: Enchytraeidae extraction v1.7

VIDEO: https://www.youtube.com/watch?v=axMicjtyoqY

A funnel extractor equipped with heat sources is used as the standard. A soil core including litter and 10 cm soil should be divided into two vertical halves (loose samples can be divided by weight). One half is used for nematode extraction, the other for enchytraeid extraction using different extraction methods (wet cold vs wet hot, gently homogenized soil vs intact soil cores, ~72 h vs 6 h extraction time). Please note that nematode extraction will not be continued during the second SBF sampling campaign. [SBF relevant] For enchytraeids:

Consumable materials:

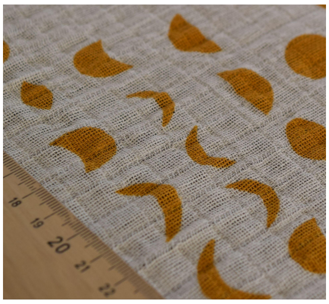

- Jute or cotton fiber* mesh with ~1 mm mesh size (20x20cm)
- Vials to store worms (2 ml glass/plastic tubes**), 1 per sample
- Ethanol ~96%, 2 ml per sample
- Printed ethanol-resistant labels, 1-2 per sample
- Drinking water, flushing bottle
- Scales, scissors, knife, spoon, tweezers, thermometer

Instructions:

1. Place required cloth on the sieves outside the funnels.
2. Place part of the soil core for enchytraeid extraction (soil and litter) on the clothes. The substrate can be divided horizontally into two parts to fill the surface of the cloth. [SBF relevant] Soil is not sieved and not homogenized!
3. Place the funnels in the funnel holders, attach the vials at the bottom of the hoses.
4. Fill the funnels with drinking water by about a half***. Check if the plugs are closed tightly. Check and remove air bubbles by squeezing the hoses.
5. Gently place the sieves with soil and litter on top of the funnels. The substrate should be fully soaked. Add more water, if needed.
6. Run the extraction with the lights (the heat source) off for 2 hours.
7. Switch the lights on and run the extraction for an additional 4 hours. The temperature in the upper part of the extractor should not exceed 40°C. Control the temperature with a thermometer.
8. When the extraction is finished (6 hours in total), strangulate the hose and gently remove the vial with worms at the bottom.
9. Discard the rest of the water from the funnel and remove the sieve and cloth.
10. Pipette very carefully ¾ of water from the top of the vial (worms are at the bottom) and fill it with 96% ethanol to fix the tissues. After the animals settle down (15 min), remove ¾ of the ethanol and refill the vial with 96% ethanol to increase concentration.
11. Put the corresponding labels in the vials and store them in a freezer. [SBF relevant]

**To have cleaner extractions, consider using one layer of muslin cloth. You can create more holes by removing some threads. If using Jute mesh, two layers are recommended.*
***Vials of other volumes can be used. Adjust the amount of ethanol and storage volume.*
****Commercial water is preferred. Tap water should be used only after 5 minutes of running and left for 24h for evaporation (remove copper and chlorine which kill worms at low concentrations). The sieve should not be of metal, except it is stainless steel.*
