# Supplementary material for: Standardized Protocols for Soil Fauna Extraction and a Call for Cross‐Lab Implementation
Source: Ecol Evol. 2026 Apr 21;16(4):e73407. doi: 10.1002/ece3.73407 (PMC13099171; doi:10.1002/ece3.73407)
Supplement: Supplementary file 3 — Data S3: Dry hot extractors. [file ECE3-16-e73407-s003.pdf]

Dry hot extractors: microarthropod extraction v1.4

VIDEO: https://www.youtube.com/watch?v=oZFEbPqHhvc

Extraction protocol using Tullgren/Berlese extractors with heating from above is presented here. Please, adapt high-gradient extraction accordingly. Extraction is done for **at least 7 days through 1 mm mesh directly into ~96% ethanol** which consequently serves as the fixing solution; no intermediate fixing agent is used.

Consumable materials:

- Vials to store microarthropods (15-20 ml tubes are recommended; it should be possible to close them tight to avoid evaporation), 1 per sample.
- Ethanol ~96%, 20-25 ml per sample.
- Printed ethanol-resistant labels, 1-2 per sample (inside and/or outside).

Instructions:

1. Place the funnels without sieves in the funnel holders.
2. Place or attach vials filled with 96% ethanol under the funnels (with labels).
3. Put soil and litter on the plastic shield on the table and leave intact or gently break into several large pieces.
4. Discard visible earthworms and large macrofauna (> 1 cm).
5. Put a sieve on another plastic shield; put 2 layers of 1 mm mesh into the sieve.
6. Put litter and soil from the first plastic shield on top of the mesh. Soil should be put upside down so animals can move through larger pore space.
7. Move the sieve with litter and soil on the first plastic shield and add soil that fell on the second plastic shield in the sieve.
8. Gently place the sieve with soil and litter on top of a funnel. Repeat for all samples.
9. Switch on the light to start the extraction.
10. Run the extraction until the sample is completely dry, but a minimum of 7 days for soil.
    a. The temperature should never exceed 50°C on the surface of the samples.
    b. Regularly check if ethanol has evaporated (after 3 days recommended) and add more if needed.
11. When the extraction is finished, remove the vials, fill them with 96% ethanol (> ⅔) and close (with labels).
12. Store all vials in a freezer (c. -20°C). [SBF relevant]
13. Remove the sieves from the funnels. Discard the soil and litter.

Important tips:

- *Animals must be able to escape down: Do not overload the funnels. There should be a maximum of 3-4 cm soil thickness on the sieve and empty spaces around the sample, allowing animals to escape laterally and down. If the funnels are small, use several funnels to extract one sample. Soil monoliths can be gently broken down.*
- *Extraction conditions should not be too harsh (e.g. too hot), because slow animals may dry out before being able to escape. Control temperature with a thermometer.*
- *Ensure appropriate extraction conditions: a ventilated room with low humidity.*
- *Sample cleanness is very important: Avoid any dirt (soil) falling in the vials. Avoid vibration, do not shake the extractors. If you check the substrate with your hands, do it above the plastic shield table, not above the extractor.*
- *Before finishing the extraction, meticulously check the sample moisture: take 2 largest samples out of the extractor and check for dryness by hand on a table. The soil should be sufficiently dry.*
